# Supplementary figures and images for: HB-EGF activates EGFR to induce reactive neural stem cells in the mouse hippocampus after seizures
Source: Life Sci Alliance. 2024 Jul 8;7(9):e202201840. doi: 10.26508/lsa.202201840 (PMC11231495; doi:10.26508/lsa.202201840)

**Fig1C**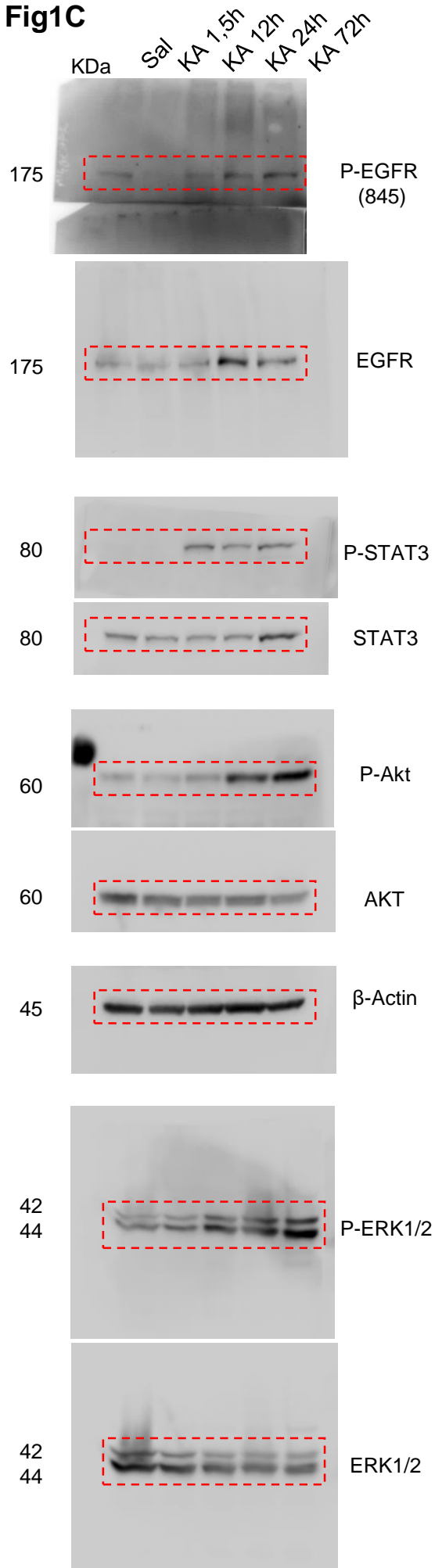**Fig2A**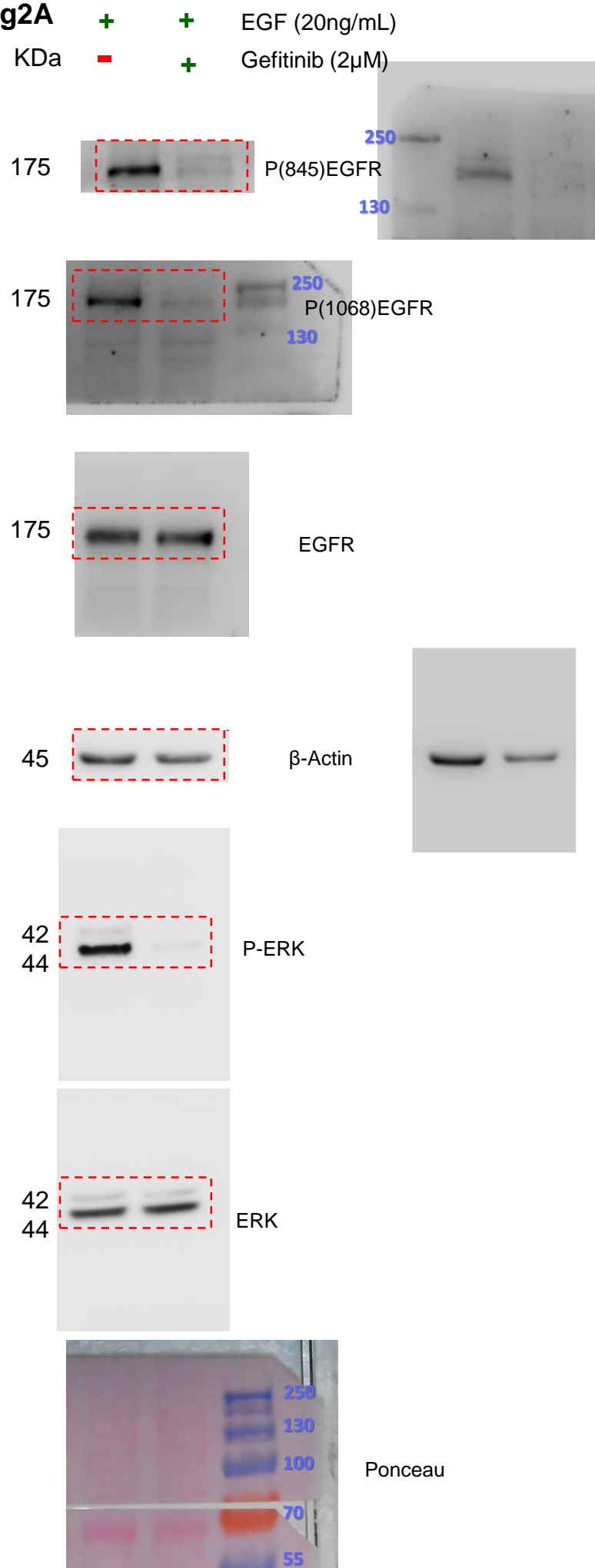

**Fig5A**

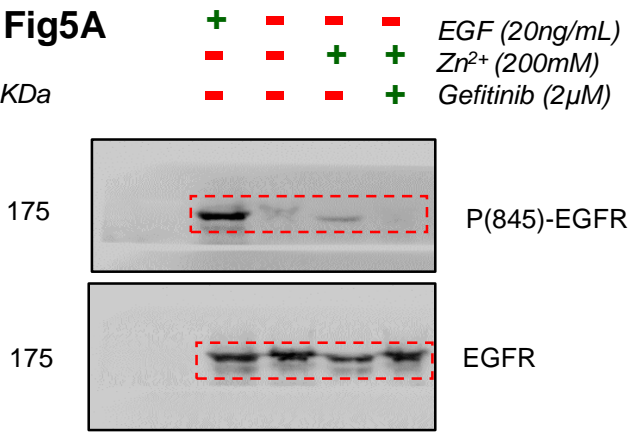

**Fig5B**

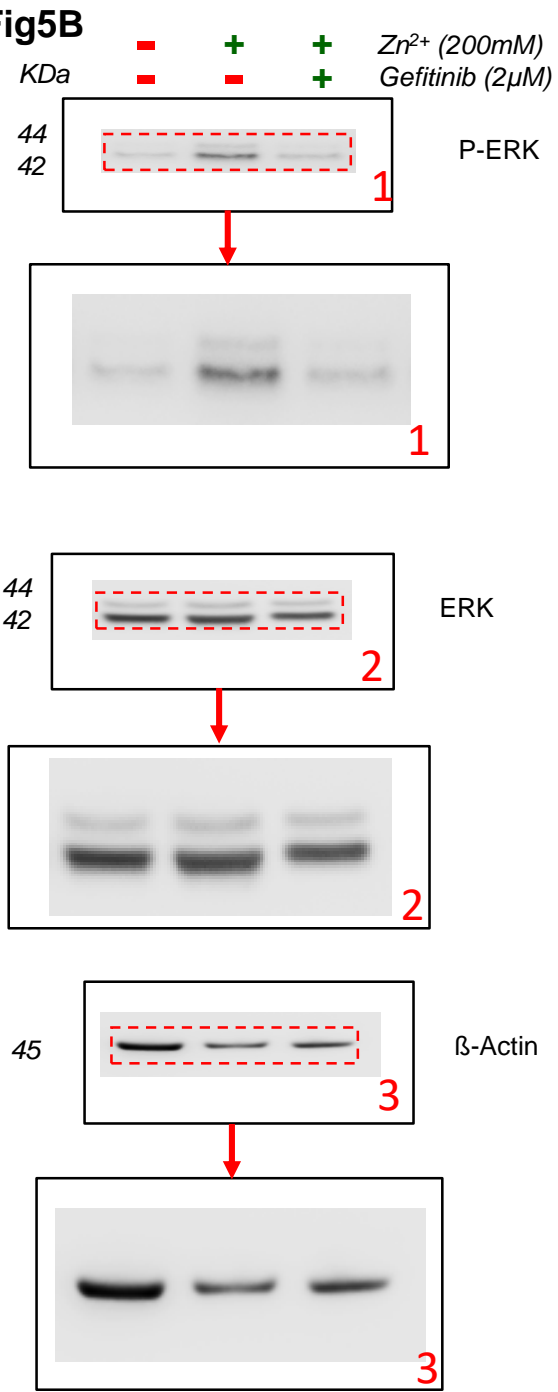

**Fig5E**

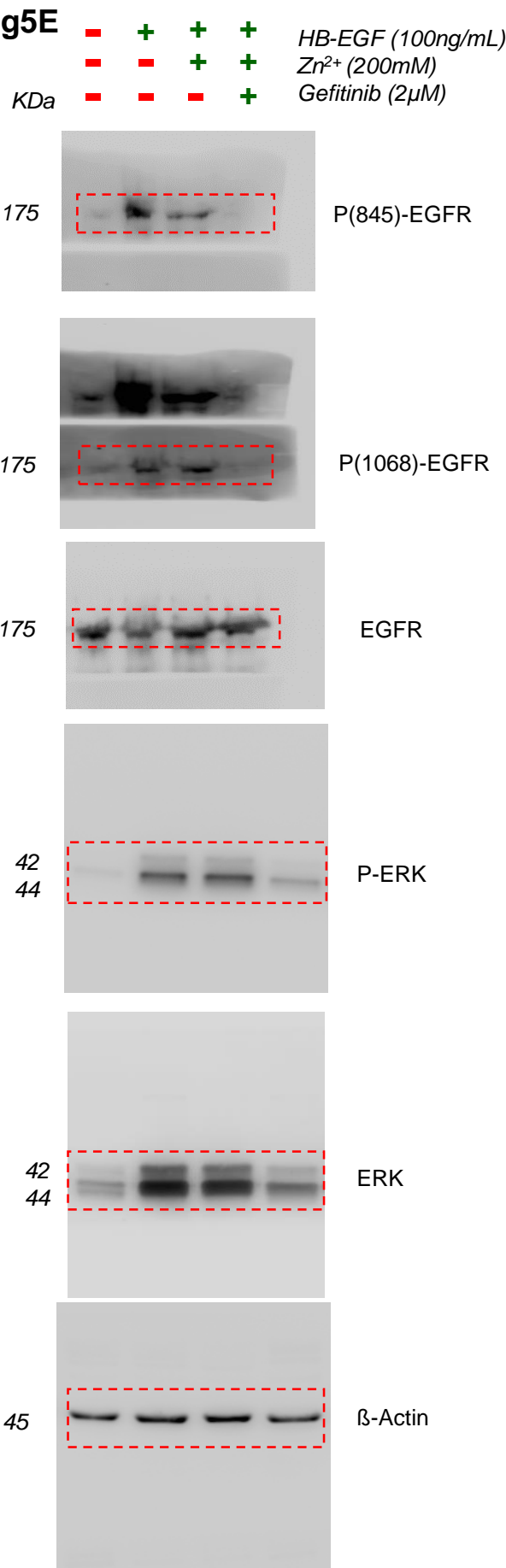

Fig S1B

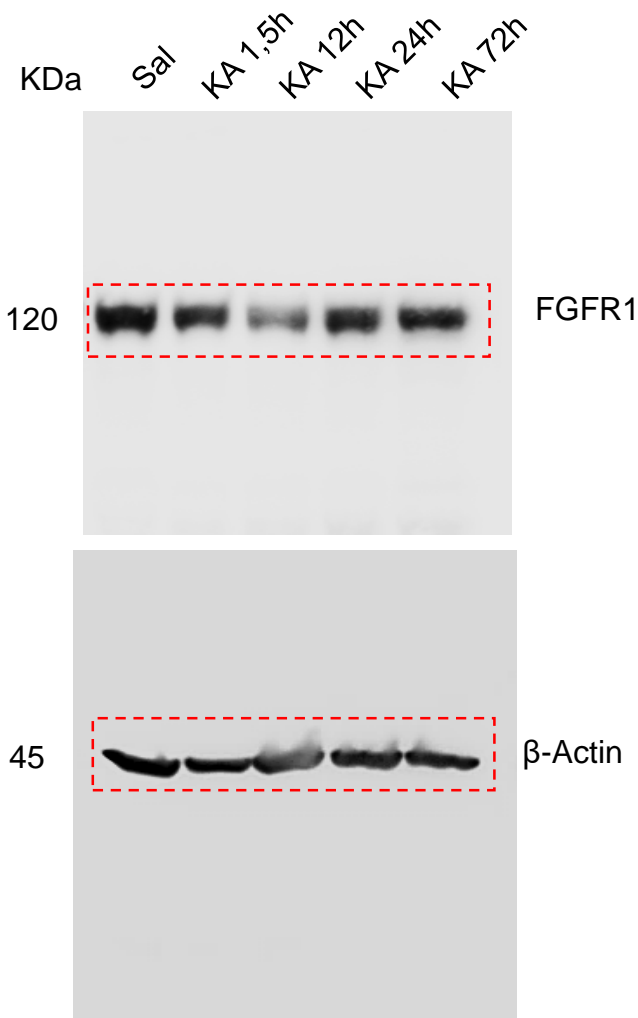

Fig S4D

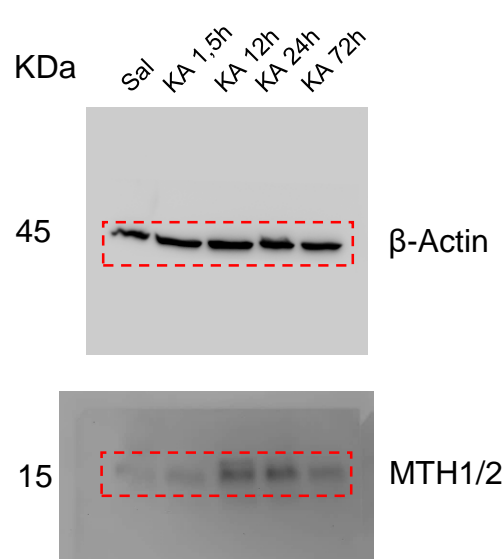

Fig S5A

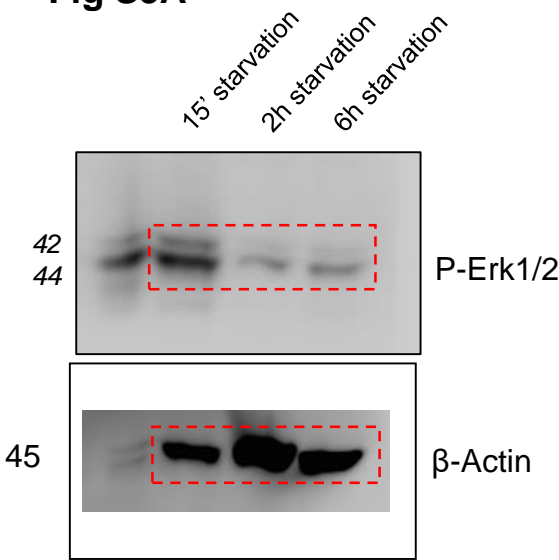

FigS5B

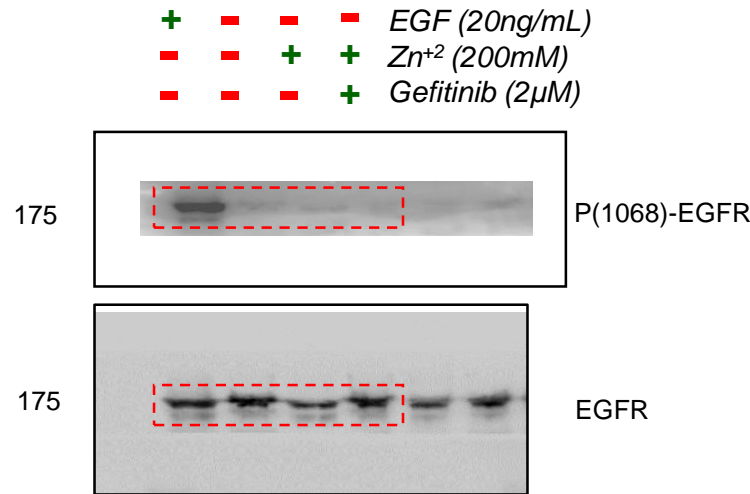

Supplement: Supplementary file 2 [file LSA-2022-01840_SdataF1.2_F2.2_F5.2_FS1.2_FS4.2_FS5.2.pdf]
